# Supplementary material for: Compounds Identified from Marine Mangrove Plant (Avicennia alba) as Potential Antiviral Drug Candidates against WDSV, an In-Silico Approach
Source: Mar Drugs. 2021 Apr 28;19(5):253. doi: 10.3390/md19050253 (PMC8145693; doi:10.3390/md19050253)
Supplement: Supplementary file 1 [file marinedrugs-19-00253-s001.zip › marinedrugs-1197646-supplementary.pdf]

Table S1: List of eleven known compound of *Avicennia alba* and their binding affinity towards to the protein generated through molecular docking method.

**Molecule PubChem CID**	**Chemical Name**	**Molecular Formula**	**Chemical Structure**	**Molecular Weight**	**Binding Affinity (kcal/mo)**
16219576	3beta-Hydroxy-20(29)-lupene	$C_{30}H_{50}O$	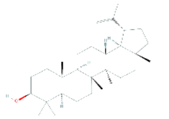	426.7 g/mol	-7.4
122130856	Lupenone	$C_{30}H_{48}O$	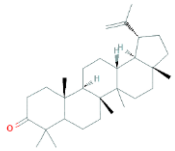	424.7 g/mol	-7.8
12303662	Phytosterols	$C_{29}H_{50}O$	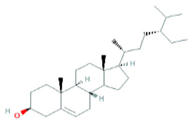	414.7 g/mol	-8.0
71597391	triterpenoids	$C_{29}H_{44}O_5$	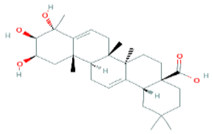	472.7 g/mol	-7.0
68972	1-Triacontanol	$C_{30}H_{62}O$		438.8 g/mol	-7.9
2371	Betulic acid	$C_{30}H_{48}O_3$	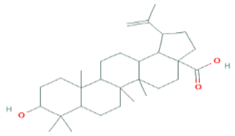	456.7 g/mol	-6.1

72326	Betulin	$C_{30}H_{50}O_2$	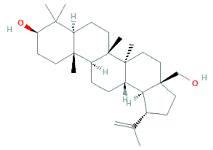	442.7 g/mol	-6.7
76419085	Bis (Acetic Acid); Tannins	$C_{80}H_{60}O_{50}$	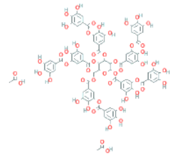	1821.3 g/mol	-3.2
244297	Friedlein	$C_{30}H_{50}O$	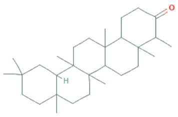	426.7 g/mol	-8.5
3884	lapachol	$C_{15}H_{14}O_3$	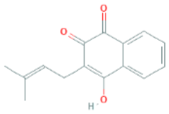	242.27 g/mol	-6.0
64945	Ursolic acid	$C_{30}H_{48}O_3$	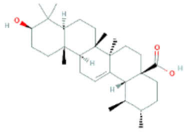	456.7 g/mol	-7.6
